# The key piece of the airway remodeling puzzle revealed**—**LIGHT signaling in the lung

**DOI:** 10.1016/j.gendis.2023.02.021

**Published:** 2023-03-27

**Authors:** Ziyan Rachel Chen, Jim Hu

**Affiliations:** a Programmes in Translational Medicine, Research Institute, Hospital for Sick Children, Toronto, Ontario M5G 0A4, Canada; b Department of Laboratory Medicine and Pathobiology, University of Toronto, Toronto, Ontario M5S 1A8, Canada; c Department of Paediatrics, University of Toronto, Toronto, Ontario M5S 1A8, Canada

Asthma is a chronic lung disease that primarily affects the lower respiratory tract in more than 300 million people worldwide. Asthma is often triggered by exposure to certain substances or conditions, such as allergens or cold air. The overreaction to the allergens causes inflammation and narrowing of the airway, which leads to episodic or persistent symptoms including shortness of breath, nocturnal cough, chest tightness, and wheezing.^1^ The cause of the disease is believed to be a combination of genetic and environmental factors. Asthma can be managed with medications that reduce inflammation and keep the airway open, such as bronchodilators and corticosteroids. However, there is currently no ideal treatment for patients with severe asthma which involves airway remodeling (AR). AR is characterized by long-term modification of the airway structure, including airway smooth muscle hyperplasia, increased inflammatory cell number, and submucosal gland hypertrophy, leading to lung function decline. Thus, understanding the molecular mechanism underlying AR is critical for the development of novel asthma therapeutics.

Recently, significant progress has been made in understanding AR development. Miki et al have successfully identified the LIGHT-LTβR non-canonical nuclear factor-κB (NF-κB) signaling pathway as the direct regulator of airway smooth muscle hyperresponsiveness (AHR) and AR pathogenesis in severe asthma.^2^ LIGHT (homologous to Lymphotoxin, exhibits Inducible expression and competes with HSV Glycoprotein D for binding to HVEM, a receptor expressed on T lymphocytes) is a cytokine that belongs to the tumor necrosis factor (TNF) family, which interacts not only with HVEM (herpes virus entry mediator) but also with LTβR (lymphotoxin β receptor).^2^ Cytokines play a crucial role in asthma as they contribute to inflammation and airway narrowing, and the TNF family has been implicated in the development and maintenance of asthma. LIGHT is primarily expressed on the surface of immune cells, including dendritic cells, neutrophils, and natural killer cells.^2^ LIGHT expression in the sputum of asthmatic patients was found to be correlated with disease severity. Previously, Croft's group has shown that LIGHT inhibition reduced AR and lung fibrosis. With LTβR knockout mice, their recent work demonstrated that AHR and airway smooth muscle (ASM) mass reduced in the absence of LTβR in an allergen-induced asthma model and that the signaling pathway involved the interaction of LIGHT-LTβR, instead of LIGHT-HVEM. In line with the theory, the introduction of exogenous LIGHT through intratracheal administration also promoted ASM hypertrophy and AHR *in vivo*.^3^ LIGHT on the surface of immune cells binds to the LTβR receptor on ASM cells, activating the non-canonical NF-κB signaling pathway and resulting in ASM hypertrophy and hyperplasia, as well as increased collagen deposition (Fig. 1).

**Figure 1:**
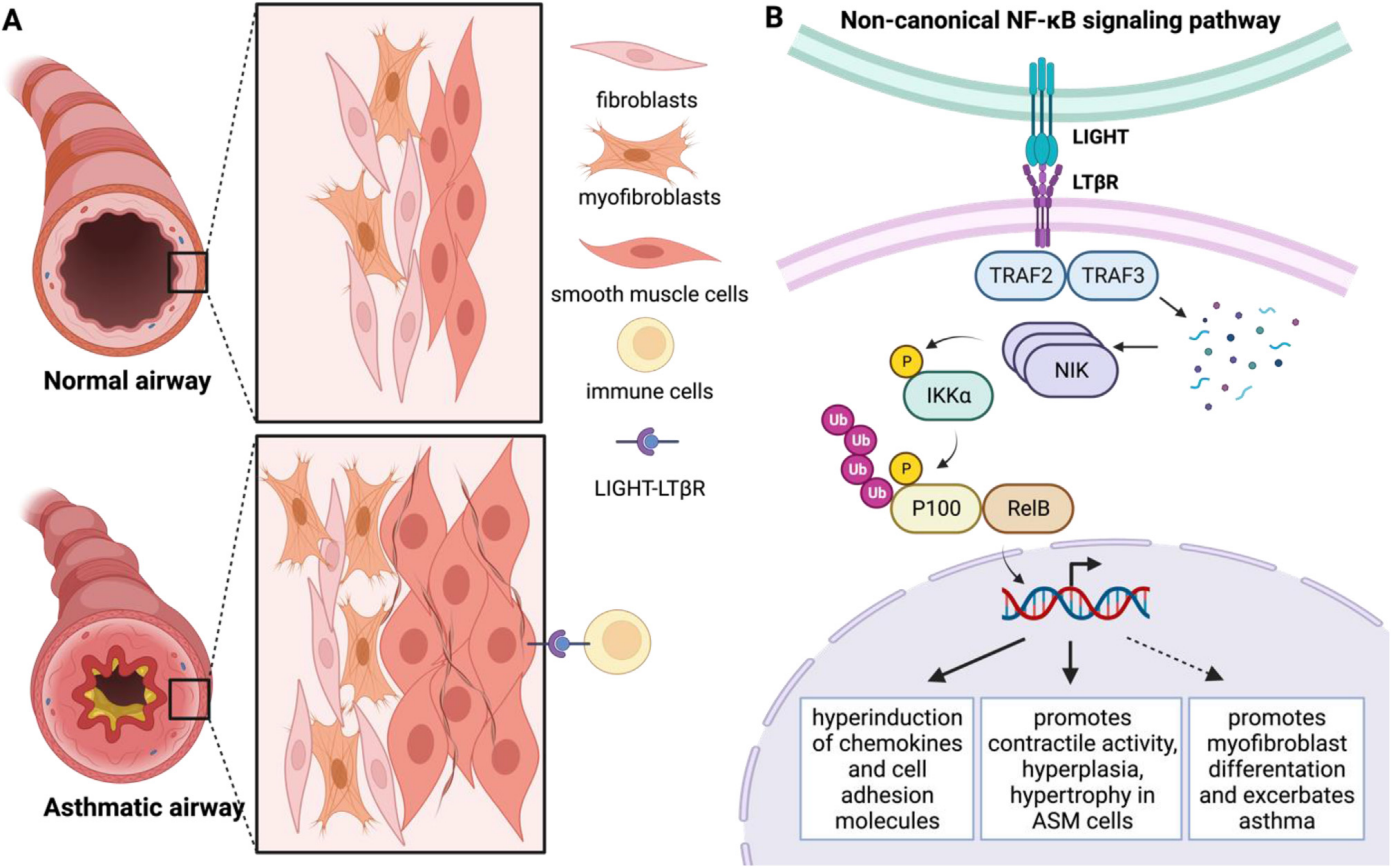
Airway remodeling mediated by LIGHT signaling pathway. **(A)** Schematic of difference between normal and asthmatic airway. Immune cells interact with smooth muscle cells through LIGHT-LTβR, activating the non-canonical NF-κB signaling pathway and resulting in ASM hypertrophy and hyperplasia. **(B)** Schematic of the non-canonical NF-κB signaling pathway in asthma. Upon LIGHT-LTβR interaction, TRAF2 and TRAF3 are recruited to the receptor. Activated TRAF2 degrades TRAF3, which allows the NF-κB inducing kinase (NIK) to accumulate. Sequentially, IKKα is induced to phosphorylate and ubiquitinate P100, leading to the nuclear translocation of RelB.^5^ As a result of the activated pathway, several events can occur, which include but are not limited to the hyper-induction of chemokines and cell adhesion molecules, ASM cell hyperplasia and hypertrophy, and potentially myofibroblast differentiation and asthma exacerbation. The figure was created with BioRender.com.

This finding shed light on potential strategies for AR targeting asthma treatment. For instance, candidates for drug screening include inhibitors that block the LIGHT-LTβR interaction or the non-canonical NF-κB signaling in ASM cells. However, more investigation is needed to validate whether AR development is dependent on the same signaling pathway in humans. In addition, apart from ASM cells, airway myofibroblasts are also implicated in AR. Myofibroblasts are essential for inflammation and repair. With characteristics of both fibroblasts and smooth muscle cells, myofibroblasts are difficult to identify due to the lack of a specific cell marker, which is also why they have been overlooked in most asthma studies. It is also known that myofibroblasts can secrete cytokines and promote AR, and can be formed from various cell types, including local fibroblasts, smooth muscle cells, and epithelial cells. ASM cells can develop a myofibroblast-like cell type when cultured *in vitro*, indicating potential myofibroblast contamination in ASM cell culture in certain *in vitro* studies.^4^ The aforementioned evidence suggests that myofibroblast's role in LIGHT-LTβR interaction or overall in asthma AR merits further examination.

## Conflict of interests

There is no conflict of interest to declare.
